# Estrone-3-Sulfate Stimulates the Proliferation of T47D Breast Cancer Cells Stably Transfected With the Sodium-Dependent Organic Anion Transporter SOAT (SLC10A6)

**DOI:** 10.3389/fphar.2018.00941

**Published:** 2018-08-21

**Authors:** Emre Karakus, Daniel Zahner, Gary Grosser, Regina Leidolf, Cemal Gundogdu, Alberto Sánchez-Guijo, Stefan A. Wudy, Joachim Geyer

**Affiliations:** ^1^Institute of Pharmacology and Toxicology, Faculty of Veterinary Medicine, Justus Liebig University Giessen, Giessen, Germany; ^2^Department of Pathology, Private Practitioner of Medicine, Erzurum, Turkey; ^3^Steroid Research and Mass Spectrometry Unit, Pediatric Endocrinology and Diabetology, Center of Child and Adolescent Medicine, Justus Liebig University Giessen, Giessen, Germany

**Keywords:** estrone-3-sulfate, T47D, breast cancer, SOAT, transport, proliferation, SLC10A6, sulfate steroid

## Abstract

Estrogens play a pivotal role in the development and proliferation of hormone-dependent breast cancer. Apart from free estrogens, which can directly activate the estrogen receptor (ER) of tumor cells, sulfo-conjugated steroids, which maintain high plasma concentrations even after menopause, first have to be imported into tumor cells by carrier-mediated uptake and then can be cleaved by the steroid sulfatase to finally activate ERs and cell proliferation. In the present study, expression of the sodium-dependent organic anion transporter SOAT was analyzed in breast cancer and its role for hormone-dependent proliferation of T47D breast cancer cells was elucidated. The SOAT protein was localized to the ductal epithelium of the mammary gland by immunohistochemistry. SOAT showed high expression in different pathologies of the breast with a clear ductal localization, including ductal hyperplasia, intraductal papilloma, and intraductal carcinoma. In a larger breast cancer cDNA array, SOAT mRNA expression was high in almost all adenocarcinoma specimen, but expression did not correlate with either the ER, progesterone receptor, or human epidermal growth factor receptor 2 status. Furthermore, SOAT expression did not correlate with tumor stage or grade, indicating widespread SOAT expression in breast cancer. To analyze the role of SOAT for breast cancer cell proliferation, T47D cells were stably transfected with SOAT and incubated under increasing concentrations of estrone-3-sulfate (E_1_S) and estradiol at physiologically relevant concentrations. Cell proliferation was significantly increased by 10^-9^ M estradiol as well as by E_1_S with EC_50_ of 2.2 nM. In contrast, T47D control cells showed 10-fold lower sensitivity to E_1_S stimulation with EC_50_ of 21.7 nM. The E_1_S-stimulated proliferation of SOAT-T47D cells was blocked by the SOAT inhibitor 4-sulfooxymethylpyrene. In conclusion: The present study clearly demonstrates expression of SOAT in breast cancer tissue with ductal localization. SOAT inhibition can block the E_1_S-stimulated proliferation of T47D breast cancer cells, demonstrating that SOAT is an interesting novel drug target from the group of E_1_S uptake carriers for anti-proliferative breast cancer therapy.

## Introduction

Estrogens play a pivotal role in the development and proliferation of hormone-dependent breast cancer, which represents the most common type of cancer in women (Conner et al., 2008). Estrogens act via nuclear ERs (Henderson et al., 1988) and the selective ER modulator tamoxifen has been used successfully for antiestrogen breast cancer therapy for four decades (Osborne, 1998). Despite a significant decline of free estrogens after menopause, a high percentage of all hormone-dependent breast cancer cases develop in this phase of life (Kenemans and Bosman, 2003). In contrast to free estrogens (E_1_ and E_2_), the sulfo-conjugated steroid forms, in particular estrone-3-sulfate (E_1_S) and DHEAS, persist at higher plasma concentrations even after menopause (Rémy-Martin et al., 1983; Geisler, 2003). These sulfo-conjugated steroids can be re-converted into active free estrogens in breast cancer tissue via cleavage of the sulfate group by the STS and further conversion by the enzymes 3β-hydroxysteroid dehydrogenase and aromatase in the case of DHEA (Santner et al., 1986; Pasqualini et al., 1997; Labrie et al., 1998; Suzuki et al., 2005; Sasano et al., 2006). Inhibitors of STS (STX64) and aromatase (anastrozole, letrozole) can block this intracrine formation of estrogens and, therefore, are used for clinical (aromatase inhibitors) or experimental (STS inhibitors) breast cancer therapy (Santen and Harvey, 1999; Stanway et al., 2007).

Prior to intracellular conversion of E_1_S and DHEAS, these negatively charged hydrophilic molecules first have to enter breast cancer cells via carrier-mediated uptake. Several uptake transporters for sulfo-conjugated steroid hormones have been characterized so far, including members of the OATP and the OAT families (Roth et al., 2012; Müller et al., 2015). Most of these OATP/OAT carriers are multi-specific and are involved in the transport of drugs, drug conjugates, bile salts, and some other charged molecules (Burckhardt, 2012; Hagenbuch and Stieger, 2013). Some of them are even expressed in breast cancer tissue or breast cancer cell lines, including OATP1A2, OATP1B3, OATP2B1, OATP3A1, OATP4A1, and others, thus making them candidates for steroid sulfate uptake in breast cancer (Pressler et al., 2011; Obaidat et al., 2012; Nakanishi and Tamai, 2014).

In the present study, we analyzed expression of the SOAT (gene name *SLC10A6*) in breast cancer. This carrier transports all physiologically occurring sulfo-conjugated steroid hormones, including E_1_S (K_m_ of 12 μM), DHEAS (K_m_ of 29 μM) and many others (Geyer et al., 2007; Fietz et al., 2013; Grosser et al., 2018). In contrast, free steroids, steroid glucuronides, or bile salts are not transported by SOAT. Therefore, this carrier can be regarded as highly specialized for sulfo-conjugated steroid hormones (Geyer et al., 2007; Grosser et al., 2018), distinguishing it from OATPs and OATs. Another difference to the OATP/OAT carriers is the fact that SOAT mediates a secondary active transport of its substrates. SOAT is highly expressed in germ cells of the testis of men and mice (Fietz et al., 2013; Grosser et al., 2013). Here, the SOAT-mediated import of sulfo-conjugated steroid hormones was suggested to participate in the overall regulation of spermatogenesis and fertility (Fietz et al., 2013; Grosser et al., 2013; Bakhaus et al., 2018). In addition, relatively high SOAT expression was detected in pancreas, placenta, and mammary gland (Geyer et al., 2007).

In the present study, high SOAT mRNA expression was found in a large set of breast cancer specimen. The SOAT protein was localized in the normal ductal epithelium of the breast and strong SOAT expression was found in breast biopsies with different pathologies. Hormone-dependent breast cancer T47D cells, stably transfected with SOAT, showed significant proliferation after incubation with E_1_S at physiologically relevant concentrations. This proliferation could be blocked successfully by SOAT inhibition, demonstrating that SOAT could be regarded as an interesting new drug target from the group of E_1_S uptake carriers for antiproliferative breast cancer therapy.

## Materials and Methods

### Materials and Chemicals

All of the chemicals, unless otherwise stated, were from Sigma-Aldrich (Taufkirchen, Germany). The compound 4-SMP was kindly provided by Prof. Dr. Hansruedi Glatt (Potsdam-Rehbrücke). [^3^H]estrone-3-sulfate ([^3^H]E_1_S, 57 Ci/mmol) was purchased from PerkinElmer (Boston, MA, United States) and [methyl-^3^H]thymidine (79 Ci/mmol) was obtained from GE Healthcare (Amersham, United Kingdom). TissueScan^TM^ breast cancer cDNA arrays I-IV (BCRT101-BCRT104) were obtained from OriGene (Rockville, MD, United States).

### Breast Cancer cDNA Arrays

In order to analyze SOAT expression in breast cancer, the following TissueScan^TM^ cDNA arrays were commercially obtained from OriGene (Rockville, MD, United States): Breast Cancer cDNA Array I (BCRT101), Breast Cancer cDNA Array II (BCRT102), Breast Cancer cDNA Array III (BCRT103), and Breast Cancer cDNA Array IV (BCRT104). Each array contains 48 samples covering tumors of different histopathology, stages and grades. For each tumor cDNA, detailed information is available online^1^, including age, gender and ethnicity of the patient, as well as diagnosis, pathology report, histologic type, tissue images, tumor grade (based on the Nottingham grading system, Elston and Ellis, 1991) and tumor stage (according to the American Joint Committee on Cancer, 2002). In addition, the receptor status is provided for the ER, PR, and HER2.

### Stable Transfection of T47D Cells With the SOAT Construct

The human breast cancer cell line T47D (obtained from Dr. Bernhard Ugele, Department of Gynecology and Obstetrics, University Hospital Munich, Germany) was maintained in a 1:1 mixture of DMEM and Ham’s F12 nutrient mixture (Invitrogen, Karlsruhe, Germany) supplemented with 10% FCS, L-glutamine (4 mM), penicillin (100 units/ml), and streptomycin (100 g/ml) at 37°C, 5% CO_2_, and 95% humidity. For stable transfection of T47D cells, the full length SOAT coding sequence (Geyer et al., 2007) was subcloned into the pcDNA3.1 vector (Invitrogen) using *Hin*dIII and *Xba*I restriction sites. The SOAT-pcDNA3 vector was verified by DNA sequencing and used for stable transfection of T47D cells by electroporation. Briefly, subconfluent T47D cells were trypsinized and resuspended in PBS containing 137 mM NaCl, 2.7 mM KCl, 1.5 mM KH_2_PO_4_, and 7.3 mM Na_2_HPO_4_ at pH 7.4. Approximately 10^6^ cells were transferred to a 4 mm Gene Pulser Cuvette (Bio-Rad Laboratories, Munich, Germany), mixed with 20 μg plasmid and incubated for 10 min on ice. The electroporation was performed on the Gene Pulser Xcell System (Bio-Rad Laboratories) using a single electrical pulse with initial field strength of 120 V, discharged from the 960 μF capacitor and time constant of 10 ms. After an additional 10 min of incubation on ice, cells were plated onto 10 cm culture dishes. After 24 h, selection medium was added containing 750 μg/ml G418 sulfate and cells were further incubated, changing the medium every 3 days. After 12 days, several cell clones were pooled from the culture dishes (T47D-SOAT) and SOAT expression was analyzed by real-time PCR analysis. For control, T47D cells were also transfected with an empty pcDNA3.1 vector. These T47D-control cells were processed in the same manner as the T47D-SOAT cells.

### Expression Analysis by Real-Time PCR

TissueScan^TM^ breast cancer cDNA arrays were directly used for expression analysis of SOAT by real-time PCR. Symplekin (SYMPK, Uniprot Q92797) that showed particularly low expression variability in breast cancer tissue and cell lines (Tilli et al., 2016) was used as endogenous control. RNA was isolated from T47D-SOAT and T47D-control cells grown in 10 cm petri dishes under DMEM/F12 medium. Cells were seeded at 10^6^ cells per well for each cell type and RNA was isolated following 72 h of growth. The medium and any detached cells were removed from the wells. Total RNA isolation was performed by using the peqGOLD RNAPure reagent (PeqLab, Erlangen, Germany) according to the manufacturer’s instructions. The isolated RNA was dissolved in diethylpyrocarbonate-treated water and stored at -80°C until use. The RNA concentration was determined by measuring absorbance at 260 nm with a Beckmann spectrophotometer DU-640 (Beckmann, Munich, Germany). Complementary cDNA was synthesized from the RNA samples using the Advantage RT-for-PCR kit (BD Clontech, Heidelberg, Germany) according to the manufacturer’s instructions. For real-time PCR expression analysis of T47D cells, beta-actin was used as endogenous control. Relative gene expression analysis was performed by real-time PCR amplification on an ABI PRISM 7300 thermal cycler (Applied Biosystems, Darmstadt, Germany) using the TaqMan Gene Expression Assays (Applied Biosystems, Darmstadt, Germany) Hs01399354_m1 for SOAT, Hs00165853_m1 for STS, Hs00174860_m1 for ERα, Hs99999903_m1 for beta-actin, and Hs00191361_m1 for SYMPK. Real-time amplification was performed in 96-well optical plates using 5 μl cDNA, 1.25 μl TaqMan Gene Expression Assay, 12.5 μl TaqMan Universal PCR Master Mix and 6.25 μl water in each 25 μl reaction. The plates were heated for 10 min at 95°C, and 45 cycles of 15 s at 95°C and 60 s at 60°C were applied. Relative expression (ΔC_T_) was calculated by subtracting the signal threshold cycle (C_T_) of the endogenous control from the C_T_ value of the respective target.

### Transport Assays in T47D Cells

For transport studies, 12-well plates were coated with poly-L-lysine for better attachment of the cells. Twenty thousand cells/well were plated and grown under DMEM/F12 medium for 3 days. Before starting the transport experiments, T47D cells were washed three times with PBS and incubated with sodium transport buffer containing 142.9 mM NaCl, 4.7 mM KCl, 1.2 mM KH_2_PO_4_, 1.2 mM MgSO_4_, 1.8 mM CaCl_2_, and 20 mM HEPES (pH 7.4). When transport assays were performed in sodium-free transport buffer, sodium chloride was substituted with equimolar concentrations of choline chloride. T47D-SOAT and T47D-control cells were incubated with 250 μl of transport buffer containing radiolabeled [^3^H]estrone-3-sulfate ([^3^H]E_1_S) at 37°C for 30 min. Transport assays were terminated by removing the transport buffer and washing five times with ice-cold PBS. Cell monolayers were lysed in 1 N NaOH with 0.1% SDS, and the cell-associated radioactivity was determined in a Wallac 1409 liquid scintillation counter (Pharmacia, Freiburg, Germany). The protein content was determined according to Lowry et al. (1951) using aliquots of the lysed cells with bovine serum albumin as a standard. LC-MS/MS was used to measure E_1_S concentrations in the cell culture medium at the end of the uptake phase as described before (Galuska et al., 2013).

### Cell Proliferation Assays in T47D Cells

T47D cells were grown for at least 1 week in DMEM/F12 supplemented with 10% FCS. Then, for proliferation assays, the cells were starved for 4 days in steroid-free phenol red-free DMEM/F12 medium supplemented with 5% dextran-coated charcoal-treated FCS (DCC-FCS). DCC-FCS was prepared by incubating 0.5 g DCC in 100 ml FCS over 24 h at 4°C, followed by filtration (pleated filter MN 615 ¼, Macherey-Nagel, Düren, Germany). T47D cells were plated at a density of 10,000 or 20,000 cells/well in 24-well plates. Twenty four hour after seeding, E_2_ (final concentration 10^-9^ M) or E_1_S (final concentrations 10^-12^ to 10^-4^ M) were added from stock solutions containing DMSO, considering that the final DMSO concentration in the medium was below 0.1%, and cells were incubated at 37°C, 5% CO_2_, and 95% humidity. The negative control included solvent alone. Seven days after seeding, cells were treated with [methyl-^3^H]thymidine with final concentrations of 1 μCi/ml at 37°C for 2 h as reported (Chalbos et al., 1982). After incubation, the medium was removed and cells were washed five times with ice-cold PBS. Then, cell lysis was performed with 500 μl of 1 N NaOH and the radioactivity of the lysates was determined by liquid scintillation counting.

### Detection of SOAT in Breast Tissues by Immunohistochemistry

Immunohistochemistry was performed on breast biopsies of different pathologies, i.e., intraductal papilloma, atypical ductal hyperplasia, intraductal carcinoma, and invasive ductal carcinoma. Paraffin-embedded tissue slides were prepared at the Department of Pathology at Atatürk University. Use of human tissue was approved by the ethics committee of Atatürk University, School of Medicine, No: 4/22, 02.06.2015. Tissue sections were incubated with the primary antibody SLC10A6 (C-13) (sc-136875, Santa Cruz, Dallas, TX, United States) at 1:100 dilution, followed by incubation with biotinylated goat anti-rabbit E0432 secondary antibody (Dako, Glostrup, Denmark) at 1:200 dilution in tris-buffered saline. Afterward, sections were incubated with the avidin-biotin complex (ABC Vectastain, Vector, Burlingame, CA, United States) and developed with 3-amino-9-ethylcarbazole (AEC, Biologo, Kronshagen, Germany). Counterstaining was performed with hematoxylin and slides were mounted with Kaiser’s glycerol gelatin (Merck, Darmstadt, Germany). Validation of the SLC10A6 C-13 antibody and the IHC protocol has been previously performed for human placenta (Schweigmann et al., 2014).

### Statistical Methods

Unless otherwise indicated, values are represented as means ± SD. All graphs and calculations were prepared using the GraphPad Prism software 6.07 (GraphPad Software, La Jolla, CA, United States). Student’s unpaired *t-*test and one-way ANOVA with Tukey’s multiple comparisons test was performed to determine statistical significance. Differences were considered significant at *p <* 0.05. The EC_50_ values were calculated by non-linear regression analysis from sigmoidal dose-response curves.

## Results

### SOAT mRNA Expression in Breast Cancer Specimen

**FIGURE 1:**
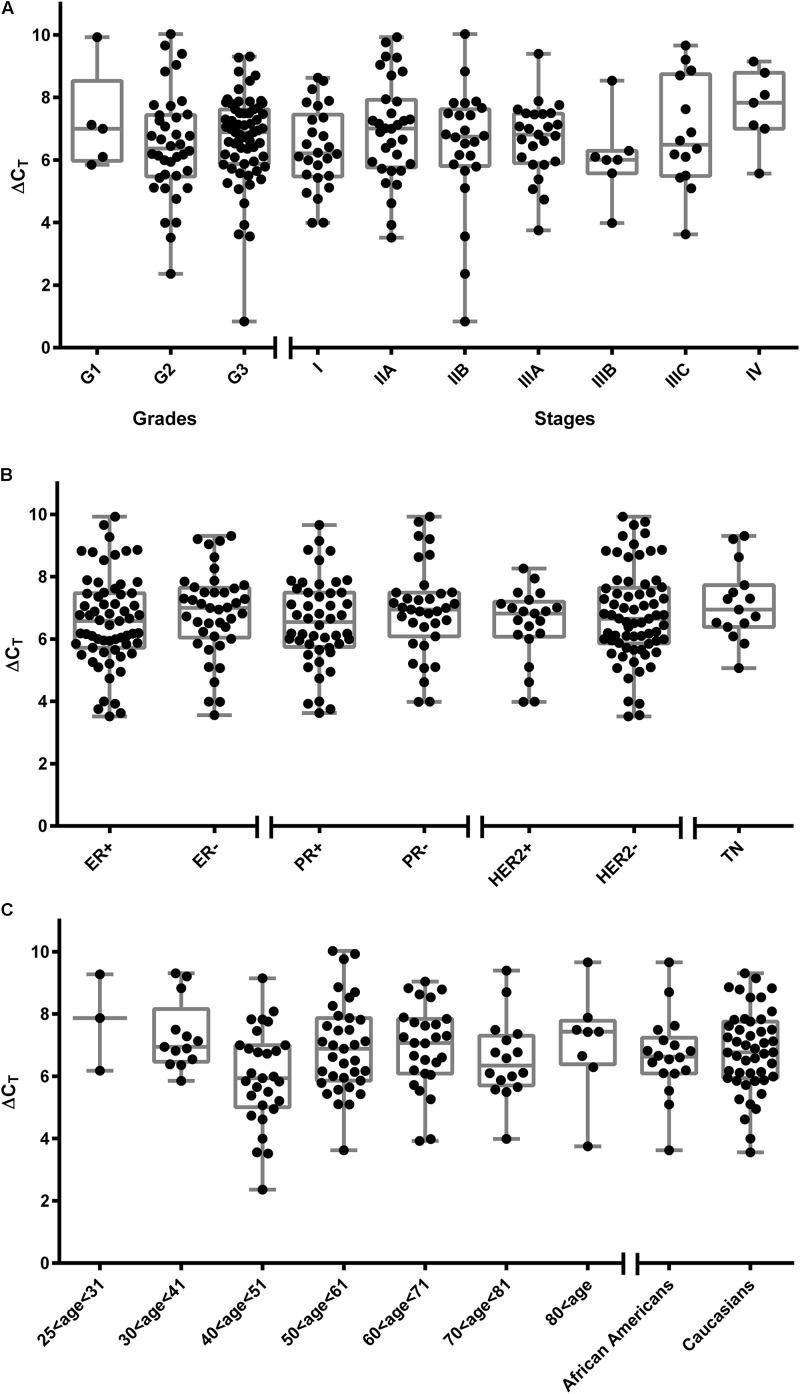
SOAT mRNA expression in breast cancer. SOAT mRNA expression was analyzed in the TissueScan^TM^ Breast Cancer cDNA Arrays I-IV, including 176 tumor cDNAs with different classifications (histopathology, grade, stage, and receptor status). Expression of SYMPK was used as endogenous control and ΔC_T_ values are depicted at the *y*-axis. A cut-off was set at C_T_ of 40. Sub-analyses were performed, including **(A)** tumor grade and stage, **(B)** receptor status for ER, PR, HER2 and triple negative breast cancer (TN), and **(C)** age and ethnos. As the cDNA arrays were not equally distributed for the analyzed subgroups, every single value is depicted for better clarity and additional box-whiskers-plots are given. For analysis of statistical significance, one-way ANOVA with Tukey’s multiple comparisons test was performed. Differences with *p* < 0.05 were not detected.

In order to analyze SOAT expression in different types of breast cancer, the OriGene TissueScan^TM^ Breast Cancer cDNA Arrays I-IV were screened for SOAT expression by real-time PCR. The arrays included 192 cDNAs from breast cancer samples of different pathology, stages, grades, and receptor status. All samples with pathology verification were included in the data analysis shown in **Figure 1**. Samples without pathology (array classification: within normal limits) were excluded from the analysis. SOAT mRNA expression was normalized by SYMPK expression, which has previously demonstrated particularly low variability of expression in breast cancer tissue and cell lines (Tilli et al., 2016). SOAT expression was undetectable only in very few samples and showed large variability in the tumor samples ranging from ΔC_T_ of 0.83 (very high expression) up to ΔC_T_ of 10 (very low expression). Nearly all tumor samples were classified as breast adenocarcinoma, with the vast majority being ductal. Only three cDNAs derived from ductal carcinoma *in situ* and one sample was from a squamous cell carcinoma of the breast. Interestingly, this squamous cell carcinoma showed extremely high SOAT expression that was even higher than in human testis, representing the organ with the highest physiological SOAT expression in man (Geyer et al., 2007; Fietz et al., 2013). In order to determine if SOAT mRNA expression correlates with tumor grade, stage, or receptor status, sub-analyses were performed. As indicated in **Figure 1A**, SOAT expression was not significantly different between tumors with grades G1, G2, or G3, or between tumors of different stages (I-IV). Furthermore, there was no difference in SOAT expression in tumors with different ER, PR, or HER2 status. Even in TN breast cancer samples, SOAT expression was not different from the other groups (**Figure 1B**). Further sub-analyses were performed in the adenocarcinoma samples including age and ethnos (**Figure 1C**). No effect of age on the SOAT mRNA expression of breast adenocarcinomas was detected and SOAT expression was comparable between Caucasians and African Americans.

**FIGURE 2:**
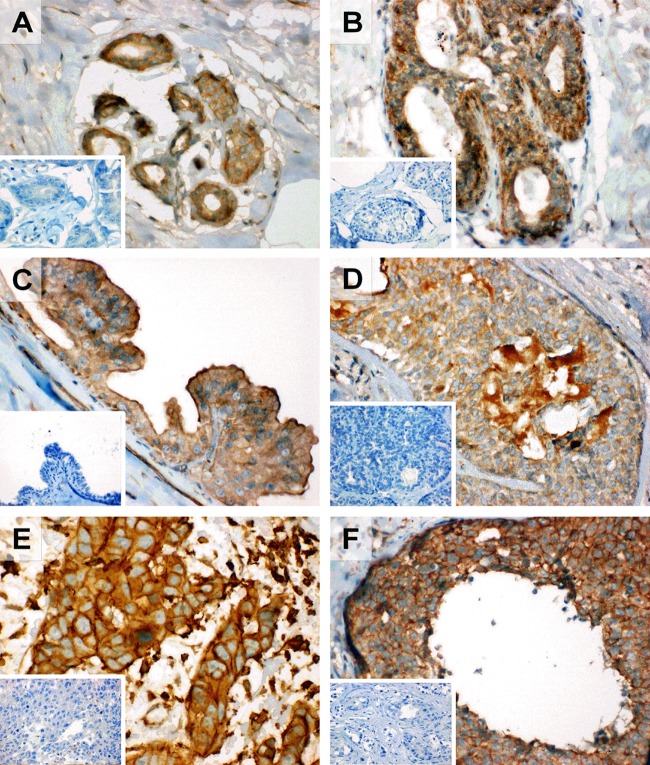
Expression of the SOAT protein in breast cancer specimen. Expression of the SOAT protein was analyzed in different breast cancer specimen by IHC with the SOAT C-13 antibody (1:100 dilution, AEC staining, hematoxylin counter stain), primary magnification × 40. Insets: Negative control without the primary antibody, primary magnification × 40. **(A)** SOAT expression in the ductal epithelium of normal breast tissue. **(B)** Strong SOAT immunoreactivity of the ductal epithelium in usual ductal hyperplasia. **(C)** Expression of SOAT in intraductal papilloma and expression along the ductal epithelium with strong apical lining. **(D)** Strong SOAT immunoreactivity in atypical ductal hyperplasia. **(E)** Severe immunolabeling with the SOAT antibody in intraductal carcinoma. **(F)** Invasive ductal carcinoma with strong and widespread SOAT expression.

SOAT expression was also analyzed in individual breast cancer samples at the protein level with the SLC10A6 (SOAT) C-13 antibody by IHC. Whereas SOAT expression was relatively low in the ductal epithelium of normal breast tissue (**Figure 2A**), strong SOAT immunoreactivity was detected in ductal hyperplasia (**Figure 2B**), intraductal papilloma (**Figure 2C**), atypical ductal hyperplasia (**Figure 2D**), intraductal carcinoma (**Figure 2E**), and invasive ductal carcinoma (**Figure 2F**).

### Generation of Stably Transfected T47D-SOAT Cells

In order to investigate the role of the carrier-mediated import of E_1_S by SOAT for the proliferation of breast cancer cells, we chose the breast cancer cell line T47D as an *in vitro* model. T47D cells have previously been described as ER expressing and they showed estrogen dependent proliferation after incubation with E_2_ and E_1_S at physiologically relevant concentrations (Nozawa et al., 2004). However, compared to breast cancer tissue (see **Figure 1**), where SOAT expression could be readily detected and quantified in nearly all specimen, SOAT expression was very low in T47D-control cells and was at the border of detectability (C_T_∼37.9) in the real-time PCR expression analysis. Therefore, in the present study, T47D cells were stably transfected with the SOAT-pcDNA3 construct in order to increase SOAT expression and mimic the situation *in vivo*. Different cell clones were pooled from the culture dishes (further referred to as T47D-SOAT). As control, T47D cells were transfected with an empty pcDNA3 vector (T47D-control). SOAT expression was analyzed by real-time PCR and revealed significantly higher mRNA expression levels in the T47D-SOAT cells compared with the T47D-control cells (ΔC_T_ = 5.4 ± 0.3 vs. ΔC_T_ = 18.0 ± 0.5). In contrast, mRNA expression levels of STS (ΔC_T_ = 10.4 ± 0.4 vs. ΔC_T_ = 9.7 ± 1.2) and ERα (ΔC_T_ = 5.2 ± 0.3 vs. ΔC_T_ = 5.5 ± 0.7) were not significantly different between T47D-SOAT and T47D-control cells.

### Transport Studies in SOAT-Transfected T47D Cells

**FIGURE 3:**
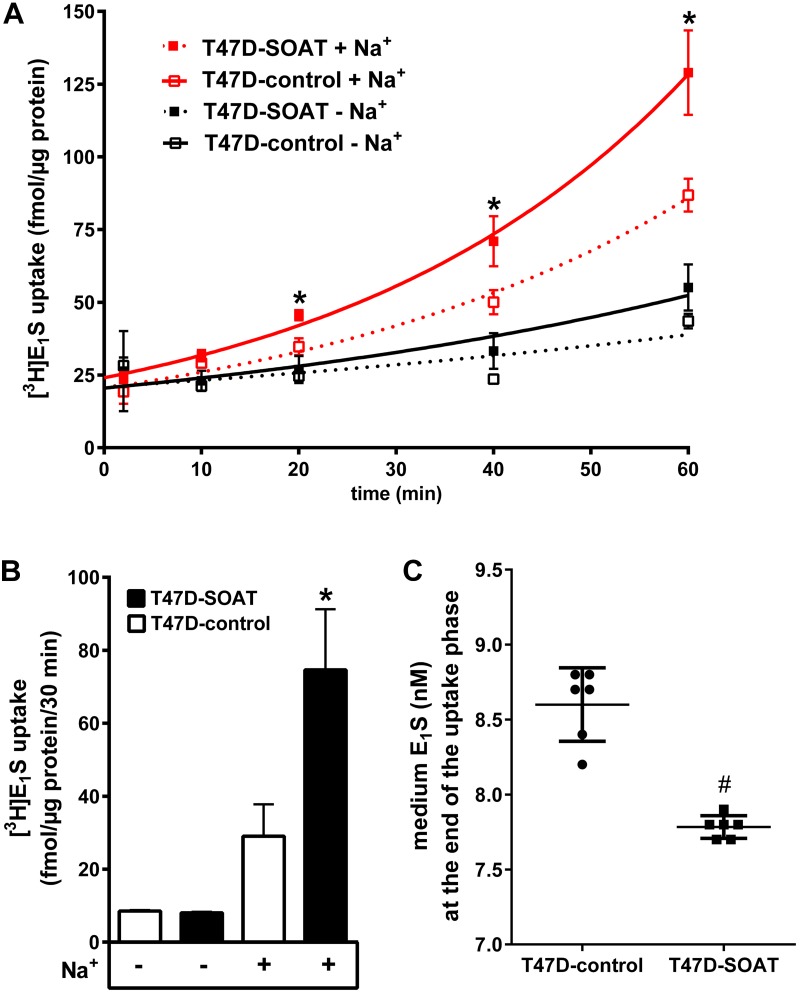
Sodium-dependent uptake of E_1_S into T47D-SOAT cells. **(A)** T47D-SOAT and T47D-control cells were incubated with 100 nM [^3^H]E_1_S at 37°C in transport buffer containing Na^+^ or without Na^+^ (sodium-free control). Cell-associated radioactivity was analyzed at the indicated time points. **(B)** Transport of [^3^H]E_1_S at 10 nM over 30 min. Data in **(A,B)** represent means ± SD of triplicate determinations. ^∗^Significantly higher transport compared with T47D-control cells and the sodium-free controls (one-way ANOVA with Tukey’s multiple comparisons test with *p* < 0.05). **(C)** Determination of E_1_S concentrations by LC-MS/MS in the cell culture medium at the end of the uptake phase. Data represent means ± SD of two independent experiments, each with triplicate determinations. ^#^Significantly different between T47D-SOAT and T47D-control cells (unpaired *t*-test with *p* < 0.05).

In order to verify functional SOAT carrier expression in the cell membrane of the T47D-SOAT cells, we performed transport experiments with [^3^H]E_1_S as substrate under both sodium and sodium-free conditions. The uptake of 100 nM E_1_S significantly increased over time in the T47D-SOAT cells only in the presence of sodium, demonstrating significant sodium-dependent uptake, which is a clear characteristic of SOAT. T47D-control cell showed slightly higher E_1_S uptake in the presence of sodium compared to sodium-free conditions, but without reaching the level of significance (**Figure 3A**). At a physiologically relevant concentration of 10 nM, T47D-SOAT cells also showed significantly higher sodium-dependent uptake of E_1_S compared with T47D-control cells (**Figure 3B**). In order to verify that under incubation with 10 nM E_1_S, this compound is indeed taken up into the cells, an additional medium depletion assay was performed and the E_1_S concentration was determined from the medium at the end of incubation by means of LC-MS/MS. As expected, the medium of the T47D-SOAT cells contained significantly lower residue concentrations of E_1_S compared to the medium of the T47D-control cells (**Figure 3C**).

### E_1_S-Stimulated Proliferation of T47D-SOAT Cells

**FIGURE 4:**
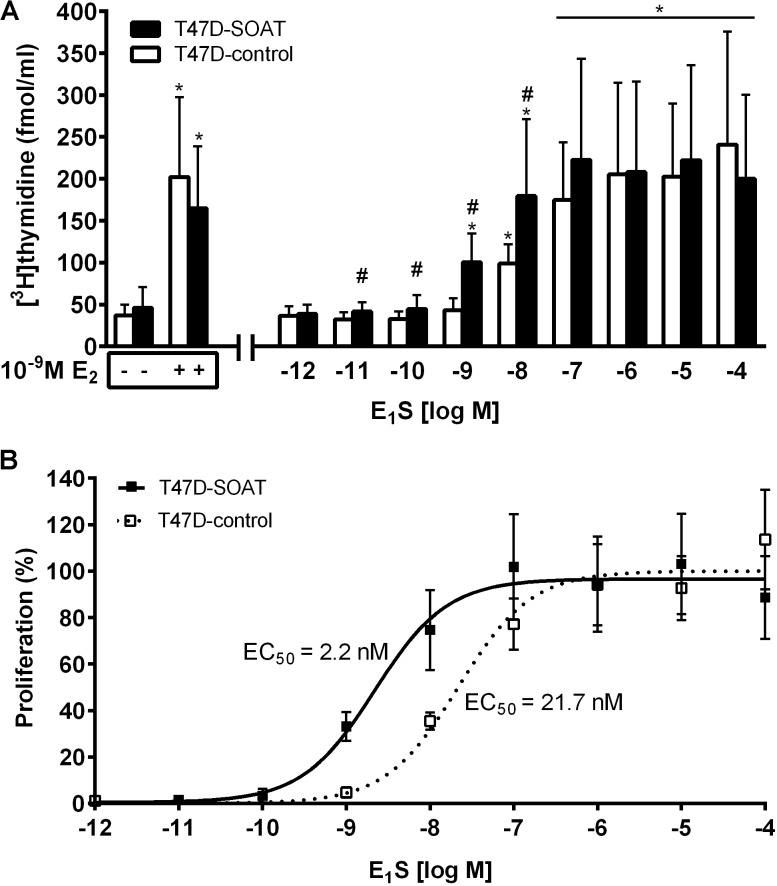
E_1_S-dependent proliferation of T47D-SOAT cells. SOAT-transfected T47D cells (T47D-SOAT, filled bars in **A**, black squares in **B**) and mock-transfected T47D cells (T47D-control, open bars in **A**, open squares in **B**) were plated at 10,000 cells/well in 24-well plates and were cultured in phenol red-free DMEM/F12 containing 5% DCC-FCS over 7 days. The culturing medium was supplemented with 10^-9^ M E_2_ for positive control or increasing concentrations of E_1_S ranging from 10^-12^ M to 10^-4^ M. For negative control, cells were treated with solvent alone. Seven days after seeding, the cells were incubated with 1 μCi/ml [^3^H]thymidine for 2 h at 37°C, 5% CO_2_, and 95% humidity. After five cycles of washing with PBS, cells were subjected to liquid scintillation counting. Data represent the means ± SD of quadruplicate determinations of three independent experiments (*n* = 12). **(A)** ^∗^Significantly higher [^3^H]thymidine incorporation compared with negative control (one-way ANOVA with Tukey’s multiple comparisons test with *p* < 0.05). ^#^Significantly higher [^3^H]thymidine incorporation in T47D-SOAT compared with T47D-control cells (*p* < 0.05, unpaired *t*-test). **(B)** Values were fitted to concentration response curves by non-linear regression analysis. Proliferation was determined relative to the top (100%) and bottom (0%) values that were derived from the sigmoidal dose response calculation. The EC_50_ values were 2.2 ± 0.3 nM and 21.7 ± 2.1 nM for the T47D-SOAT and T47D-control cells, respectively. Data represent means ± SEM.

In order to analyze the estrogen-dependent proliferation of the transfected cell lines, T47D-SOAT and T47D-control cells were grown in DCC-FCS medium supplemented with 10^-9^ M E_2_ (positive control), increasing concentrations of E_1_S ranging from 10^-12^ M to 10^-4^ M, or solvent alone (negative control). Both cell lines showed significantly increased proliferation under E_2_ treatment and this proliferation occurred at equal levels for the SOAT-T47D and SOAT-control cells (**Figure 4A**). After treatment with E_1_S at increasing concentrations, both cell lines showed increased proliferation, but with different profiles. Beginning at 10^-11^ M E_1_S, T47D-SOAT cells showed significantly enhanced proliferation compared to T47D-control cells with a maximum proliferation at 10^-7^ M. In contrast, T47D-control cells did not start to proliferate until 10^-9^ M E_1_S and required 10^-6^ M E_1_S for maximum proliferation (**Figure 4A**). Within the concentration range of 10^-11^ to 10^-8^ M E_1_S, T47D-SOAT showed significantly higher proliferation compared to T47D-control cells. Half-maximal stimulation (ED_50_) of the E_1_S-stimulated proliferation occurred at concentrations of 2.2 nM and 21.7 nM for the T47D-SOAT and T47D-control cells, respectively (**Figure 4B**). This indicates that SOAT-mediated transport of E_1_S at physiologically relevant concentrations significantly stimulated the proliferation of T47D cells.

**FIGURE 5:**
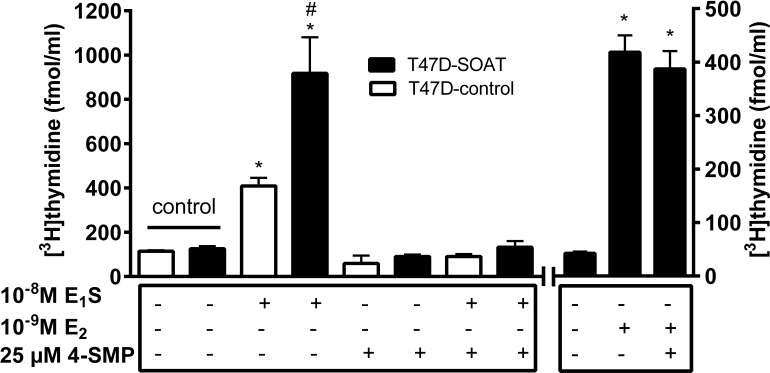
Inhibition of the E_1_S-dependent proliferation of T47D-SOAT cells. Stably SOAT-transfected T47D cells (T47D-SOAT) and mock-transfected T47D cells (T47D-control) were plated at 20,000 cells/well in 24-well plates and were cultured in phenol red-free DMEM/F12 containing 5% DCC-FCS. The culturing medium was supplemented with 10^-8^M E_1_S, 10^-9^ M E_2_ or solvent alone (control). Additionally, 25 μM of the SOAT inhibitor 4-SMP were added to the culturing medium as indicated. After 6 days of cultivation, the cells were incubated with 1 μCi/ml [^3^H]thymidine for 2 h at 37°C, 5% CO_2_, and 95% humidity. After five cycles of washing with PBS, cells were subjected to liquid scintillation counting. Data represent means ± SD of quadruplicate determinations of a representative experiment. ^∗^Significantly different from solvent control (one-way ANOVA with Tukey’s multiple comparisons test with *p* < 0.05). ^#^Significantly higher [^3^H]thymidine incorporation in T47D-SOAT compared with T47D-control cells (*p* < 0.05, unpaired *t*-test).

In order to block this increased proliferation via SOAT, 4-SMP was used as an inhibitor. 4-SMP proved to be one of the most potent inhibitors of SOAT in a previous study (Geyer et al., 2007). Indeed, incubation of T47D-SOAT cells with 25 μM 4-SMP completely blocked cell proliferation by E_1_S (**Figure 5**), while 4-SMP alone had no effect on the cell proliferation of T47D-SOAT and T47D-control cells. Furthermore, 4-SMP had no effect on the E_2_-stimulated cell proliferation of T47D-SOAT cells, which occurs independent from SOAT-mediated transport (**Figure 5**, right panel).

**FIGURE 6:**
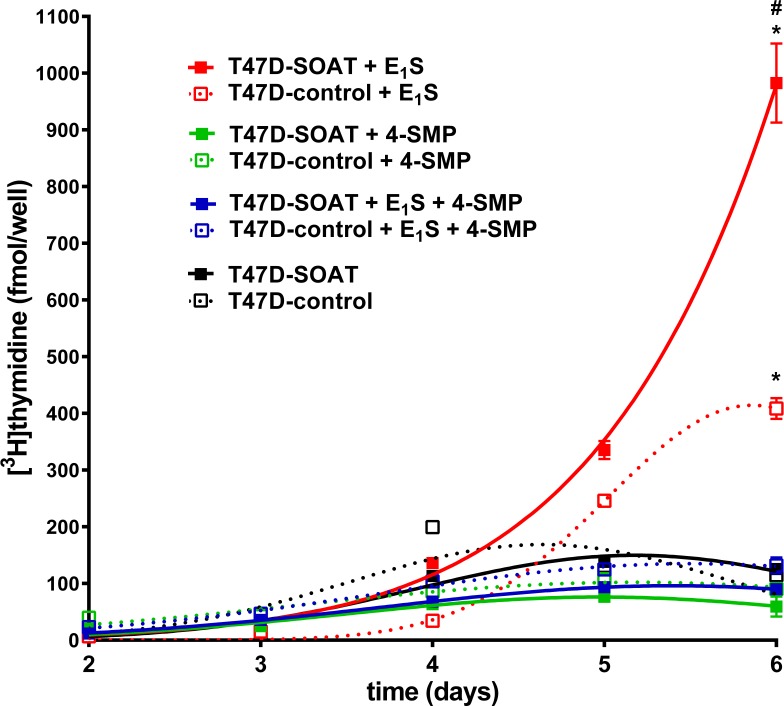
Time course of the E_1_S-dependent proliferation of T47D-SOAT cells. T47D-SOAT and T47D-control cells were plated at 20,000 cells/well in 24-well plates and were cultured in phenol red-free DMEM/F12 containing 5% DCC-FCS. The culturing medium was supplemented with 10^-8^ M E_1_S, 25 μM 4-SMP or solvent alone. Additionally, 10^-8^ M E_1_S were incubated together with the SOAT inhibitor 4-SMP at 25 μM. After 2, 3, 4, 5, and 6 days of cultivation, the cells were incubated with 1 μCi/ml [^3^H]thymidine for 2 h at 37°C, 5% CO_2_, and 95% humidity. After five cycles of washing with PBS, cells were subjected to liquid scintillation counting. Data represent the means ± SD of quadruplicate determinations of a representative experiment. ^∗^Significant increase compared to day 2 (*p* < 0.05, one-way ANOVA with Tukey’s multiple comparisons test). ^#^Significantly higher [^3^H]thymidine incorporation in T47D-SOAT compared to T47D-control cells (*p* < 0.05, unpaired *t*-test).

In further experiments, the time course of E_1_S-stimulated T47D cell proliferation was analyzed in further detail with and without 4-SMP as inhibitor of SOAT. Both cell lines, T47D-SOAT and T47D-control, significantly increased their proliferation from days 4 to 6 under incubation with 10 nM E_1_S, but at day 6, T47D-SOAT cells showed significantly higher [^3^H]thymidine incorporation compared with the T47D-control cells. In cells additionally incubated with 4-SMP, proliferation was blocked and was not different from the control groups without E_1_S incubation (**Figure 6**). Again, 4-SMP alone had no effect on cell proliferation.

## Discussion

As has been known for a long time, breast tumor tissue is able to metabolize steroids from sulfated precursors, which are available even after menopause, to biologically active estrogens (Adams and Wong, 1968; Miller and Forrest, 1976). Most studies in this field focus on the metabolic steps involved in estrogen formation, and simply presume intracellular availability of the mostly sulfated precursors (Labrie et al., 2001). However, sulfo-conjugated steroids are negatively charged molecules and can only enter target cells via carrier-mediated uptake (Müller et al., 2015). Therefore, several previous studies have investigated the expression of uptake carriers for sulfated steroids in the normal mammary gland and breast cancer tissue, including OATP1A2, OATP2B1, OATP3A1, OATP4A1, and OATP1B3 (Pizzagalli et al., 2003; Miki et al., 2006; Meyer zu Schwabedissen et al., 2008; Wlcek et al., 2008; Kindla et al., 2011; Banerjee et al., 2014). Furthermore, E_1_S uptake carrier expression was demonstrated in different hormone-dependent breast cancer cell lines, such as OATP1A2, OATP3A1, and OATP4A1 in T47D cells (Nozawa et al., 2004, 2005; Meyer zu Schwabedissen et al., 2008) as well as OATP1A2, OATP2B1, OATP3A1, and OATP4A1 in MCF7 cells (Nozawa et al., 2005; Meyer zu Schwabedissen et al., 2008; Wlcek et al., 2008; Maeda et al., 2010; Stute et al., 2012; Banerjee et al., 2012, 2013, 2015; Matsumoto et al., 2015). Overall these studies demonstrated that carrier-mediated import of E_1_S in breast cancer cells can stimulate their proliferation and thus these carriers were suggested as potential drug targets (Obaidat et al., 2012; Nakanishi and Tamai, 2014).

In the present study, we only focused on the expression of SOAT in breast cancer and aimed to analyze its role for hormone-dependent proliferation. SOAT was first cloned from rat adrenal gland and demonstrated significant transport of E_1_S and DHEAS (Geyer et al., 2004). Later, the human SOAT transcript was cloned (Geyer et al., 2007). The substrate spectrum of SOAT was intensively analyzed and revealed specific transport of all physiologically occurring sulfo-conjugated steroid hormones, being E_1_S, 17β-estradiol-3-sulfate, 17β-estradiol-17-sulfate, pregnenolone sulfate, 17α-OH-pregnenolone sulfate, androsterone sulfate, epiandrosterone sulfate, testosterone sulfate, epitestosterone sulfate, 5α-dihydrotestosterone sulfate, androstenediol sulfate, DHEAS, and 16α-OH-DHEAS (Geyer et al., 2007; Fietz et al., 2013; Schweigmann et al., 2014; Grosser et al., 2018). Sulfo-conjugated bile salts, bromosulfophthalein (BSP, a dye used in liver function tests), and the sulfooxymethylpyrenes 2-SMP and 4-SMP were identified as effective SOAT inhibitors (Geyer et al., 2007). Apart from high SOAT expression in the testis, placenta, lung and skin, SOAT also showed relatively high mRNA expression in the mammary gland (Geyer et al., 2007). Here, in the present study the SOAT protein was localized to the ductal epithelium (**Figure 2**). Based on this data, it was not surprising that SOAT showed high expression in different pathologies of the breast with a clear ductal staining pattern in IHC, including ductal hyperplasia, intraductal papilloma and intraductal carcinoma (**Figure 2**). It can be supposed that, in addition to other E_1_S carriers from the OATP family, SOAT expression in ductal hyperplasia and intraductal carcinoma contributes to the import of E_1_S (and probably also E_2_S) and therefore provides the precursors for the intracrine formation of pro-proliferative E_2_. Based on this mechanism, SOAT can be regarded as an additional drug target for anti-proliferative breast cancer therapy from the group of E_1_S uptake carriers. Against this background, it is interesting to note that SOAT expression was detected in a wide range of breast cancer specimen. Most of them represent adenocarcinoma of the breast with ductal localization (**Figure 1**). SOAT expression does not correlate with tumor stage, grade or receptor status. Therefore, SOAT might be an interesting drug target in a wide variety of breast tumors.

In order to verify the role of SOAT for the E_1_S-dependent proliferation of breast cancer cells, we intended to generate an *in vitro* model that mimics the *in vivo* situation as near as possible. Different ER positive breast cancer cell lines such as T47D and MCF7 have emerged as *in vitro* models for proliferation studies in the past (MacIndoe, 1988; Pasqualini et al., 1990; Evans et al., 1993; Billich et al., 2000; Schmitt et al., 2001; Maggiolini et al., 2001, 2002). Of those, we used T47D cells, as their intracrine estrogen synthesis is well characterized and E_1_S uptake carrier expression has been investigated previously on these cells (Pizzagalli et al., 2003; Nozawa et al., 2004; Miki et al., 2006). However, as demonstrated by real-time PCR, SOAT expression seems to be down-regulated in T47D cells compared to breast cancer tissue. Therefore, we generated a SOAT-transfected T47D-SOAT cell line as well as a mock-transfected T47D-control cell line for proliferation assays. In the T47D-SOAT cells, high SOAT mRNA expression was confirmed by real-time PCR at the transcriptional level as well as by significant sodium-dependent transport of [^3^H]E_1_S at the level of the active transporter protein (**Figure 3**). For proliferation studies, these T47D-SOAT cells were incubated under increasing concentrations of E_1_S at 10^-12^ to 10^-4^M. As previously described (Nozawa et al., 2004), proliferation of T47D cells can be stimulated not only by E_2_, but also by E_1_S at higher concentrations. In the present study, we obtained a sigmoidal dose-response curve with an effective concentration (EC_50_) of 21.7 nM for the T47D-control cells, which is very close to previous data, being 17.1 nM (Nozawa et al., 2004). Interestingly, proliferation of the SOAT-expressing T47D-SOAT cells was stimulated already at much lower E_1_S concentrations and the dose-response curve revealed an EC_50_ of 2.2 nM, meaning a 10-fold increased sensitivity to E_1_S exposure. At physiological E_1_S concentrations, being in the order 10^-8^ to 10^-9^ M (Pasqualini, 2004), the differences between the proliferations of T47D-SOAT vs. T47D-control cells were most pronounced (**Figure 4**). To ensure that this increased proliferation is indeed induced by SOAT-mediated import of E_1_S, the cells were co-incubated with E_1_S and the SOAT inhibitor 4-SMP (Geyer et al., 2007). In these experiments, E_1_S was added at 10 nM to stimulate cell proliferation, representing a concentration at which T47D-SOAT cells showed significant sodium-dependent transport activity for [^3^H]E_1_S. Interestingly, 4-SMP completely blocked the E_1_S-mediated proliferation of T47D-SOAT cells and likewise that of T47D-control cells. To verify that 4-SMP itself does not cause inhibition of proliferation by any other effect than by inhibiting the E_1_S uptake, 4-SMP was additionally co-incubated with E_2_. In these experiments, the E_2_-stimulated proliferation of the T47D-SOAT cells was not affected by 4-SMP, indicating that 4-SMP had no effect on the direct estrogenic effect of E_2_, but only on the transport of E_1_S. The inhibiting effect of 4-SMP on the T47D-control cells could mean that here, residue E_1_S transport activity is inhibited even if SOAT is expressed at very low levels in the T47D-control cells. Another explanation would be that 4-SMP inhibits other E_1_S uptake transporters, such as OATP1A2, OATP2B1, OATP3A1, and OATP4A1, which have been detected previously in T47D cells (Pizzagalli et al., 2003; Nozawa et al., 2004; Miki et al., 2006). At least in the case of OAT3, 4-SMP has been described as inhibitor (Bakhiya et al., 2006), indicating that 4-SMP is not selective for SOAT. However, if 4-SMP does interfere with the E_1_S transport via one of the mentioned OATPs must be elucidated.

## Conclusion

The present study demonstrates expression of SOAT in breast cancer tissue with ductal localization. SOAT inhibition can block the E_1_S-stimulated proliferation of T47D breast cancer cells and, therefore, in addition to the carriers of the OATP carrier family, can be regarded as a novel potential drug target for anti-proliferative breast cancer therapy. Very recently, novel inhibitors of SOAT were identified by pharmacophore modeling (Grosser et al., 2016). After optimization, these compounds are interesting candidates for further breast cancer proliferation studies *in vitro* and *in vivo*. These studies should also include comparative transport inhibition of SOAT and carriers of the OATP and OAT families in order to find at best an inhibitor for E_1_S transport covering all uptake carriers in breast cancer cells.

## Author Contributions

EK, GG, RL, CG, and AS-G acquired and analyzed the data. DZ, SW, and JG interpreted the data and wrote the manuscript. DZ and JG conceived, designed, and supervised the study.

## Conflict of Interest Statement

The authors declare that the research was conducted in the absence of any commercial or financial relationships that could be construed as a potential conflict of interest.

## Footnotes

1 https://www.origene.com/products/tissues/tissuescan

## Abbreviations

4-SMP: 4-sulfooxymethylpyrene
DCC: dextran-coated charcoal
DHEAS: dehydroepiandrosterone sulfate
DMEM: Dulbecco’s Modified Eagle’s Medium
DMSO: dimethyl sulfoxide
E_1_: estrone
E_2_: estradiol
E_1_S: estrone-3-sulfate
ER: estrogen receptor
FCS: fetal calf serum
HER2: human epidermal growth factor receptor 2
IHC: immunohistochemistry
LC-MS-MS: liquid chromatography-tandem mass spectrometry
OAT: organic anion transporter
OATP: organic anion transporting polypeptide
PBS: phosphate buffered saline
PR: progesterone receptor
SOAT: sodium-dependent organic anion transporter
STS: steroid sulfatase
TN: triple negative
